# Pharmacokinetics of Local Anesthetics in Fascial Plane Blocks: Clinical Implications and Safety Considerations-A Narrative Review

**DOI:** 10.4274/TJAR.2026.262464

**Published:** 2026-10-30

**Authors:** Musa Zengin, Hadi Ufuk Yörükoğlu, Oğuz Gündoğdu, Aslıhan Aykut, Emine Nilgün Zengin, Cemre Tokuş, Elif Şule Özdemir Sezgi, Onur Küçük

**Affiliations:** 1 University of Health Sciences Türkiye, Ankara Etlik City Hospital, Clinic of Anaesthesiology and Reanimation, Ankara, Türkiye; 2 Kocaeli University Faculty of Medicine, Department of Anaesthesiology and Reanimation, Kocaeli, Türkiye; 3 Sivas Cumhuriyet University Faculty of Medicine, Department of Anaesthesiology and Reanimation, Sivas, Türkiye; 4 Ankara Bilkent City Hospital, Clinic of Anaesthesiology and Reanimation, Ankara, Türkiye; 5 Dokuz Eylül University Faculty of Medicine, Department of Pharmacology, İzmir, Türkiye; 6 Dokuz Eylül University Faculty of Medicine, Department of Anaesthesiology and Reanimation, İzmir, Türkiye

**Keywords:** Fascial plane blocks, local anesthetics, adjuvants, local anesthetic systemic toxicity, pharmacokinetics, regional anaesthesia

## Abstract

Fascial plane blocks (FPBs) are increasingly used in modern regional anaesthesia and differ from conventional nerve blocks by delivering local anesthetic (LA) into interfascial compartments. This approach results in distinct pharmacokinetics with clinically relevant systemic absorption. Pharmacokinetic behavior in FPBs is determined by interactions among patient-related factors, drug properties, adjuvant use, and fascial plane characteristics. High-volume injections, tissue vascularity, and injection technique influence peak plasma concentration and time to peak concentration. Patient variables, such as advanced age, pregnancy, obesity, malnutrition, and organ dysfunction, further increase variability and the risk of toxicity. Epinephrine may reduce the absorption rate and improve safety, whereas dexamethasone and dexmedetomidine mainly prolong analgesia through pharmacodynamic effects. Because systemic absorption is inherent to FPBs, LA systemic toxicity remains a key concern. Individualized dosing, appropriate drug selection, and close monitoring are essential for safe and effective clinical practice.

**Main Points**

• Fascial plane blocks (FPBs) exhibit distinct pharmacokinetics characterized by high-volume interfascial spread and clinically relevant systemic absorption, making peak plasma concentration and time to peak critical determinants of safety.

• Pharmacokinetic variability is driven by the interplay between patient factors, drug properties, adjuvants, and fascial plane anatomy, necessitating individualized dosing strategies.

• Systemic exposure after FPBs may approach or exceed toxicity thresholds even within conventional dose limits, highlighting the persistent risk of local anesthetic systemic toxicity.

• Rational selection of drugs and adjuvants, combined with vigilant monitoring, is essential for optimizing analgesic efficacy while maintaining pharmacokinetic safety.

## Introduction

Fascial plane blocks (FPBs) involve the injection of local anesthetic (LA) into potential interfascial compartments rather than around a discrete peripheral nerve. The ability of deep fascial layers to form expandable interfascial spaces provides the anatomical basis for these techniques and facilitates the spread of injectate over relatively large tissue planes.^1, 2^ Although ultrasound guidance enables visualization of fascial separation and injectate spread, visible distribution does not necessarily predict sensory nerve coverage. Effective analgesia is thought to depend, at least in part, on adequate sensory nerve coverage within the surgical field.^3^

Injectate spread within fascial planes is dynamic and may extend beyond the initial ultrasound image over time, potentially increasing sensory nerve coverage.^1^ This property may underlie the volume-dependent nature of FPBs, whereby increasing injectate volume increases sensory nerve coverage and thereby contributes to variability in clinical effects.^4^ However, volume dependence also introduces variability, contributing to inconsistencies in dermatomal sensory mapping and clinical effects.

Pharmacokinetically, interfascial injections differ from traditional nerve blocks by distributing LA over extended tissue planes rather than confining it within a discrete compartment. This pattern facilitates systemic absorption, as demonstrated by measurable plasma concentrations following truncal FPBs.^5^ Variability in peak plasma concentration (C_max_) and time-to-peak concentration (T_max_) across techniques suggests that anatomical plane characteristics and injection variables significantly influence systemic exposure.^5^ Since systemic toxicity is closely related to circulating LA concentrations, understanding these determinants is important for safe clinical practice.^6^

With the rapid expansion of FPBs in modern regional anaesthesia, their clinical performance is increasingly linked to pharmacokinetic determinants, including absorption, plasma concentration profiles, duration of action, and safety margins.^7^ This review synthesizes current evidence on patient-related characteristics, LA properties, adjuvant use, and fascial plane anatomy as key determinants of pharmacokinetic variability in FPBs and provides a structured, clinically applicable framework to optimize analgesic efficacy while minimizing systemic exposure and the risk of LAST.

### Methods of the Search Strategy

This narrative review was informed by literature searches conducted in March 2026 using MEDLINE/PubMed, Embase, the Cochrane Central Register of Controlled Trials, Google Scholar, and Scopus. To ensure that the review reflected the most recent evidence, the literature search was updated prior to finalizing the manuscript to include publications available up to May 2026. Search strategies included combinations of the terms “fascial plane block,” “interfascial plane block”, “pharmacokinetics,” “plasma concentration,” “serum concentration,” “peak plasma,” “local anesthetic,” “lidocaine,” “bupivacaine,” “levobupivacaine,” “liposomal bupivacaine,” “prilocaine,” “ropivacaine,” “adjuvant,” “epinephrine,” “dexmedetomidine,” “dexamethasone,” “magnesium sulfate,” “opioid,” “local anesthetic systemic toxicity,” “erector spinae plane block,” “ESP block,” “ESPB,” “rectus sheath block,” “transversus abdominis plane block,” “TAP block,” “quadratus lumborum block,” “QL block,” “interpectoral plane block,” “pectoserratus plane block,” “PECS block,” “serratus anterior plane block,” “SAP block,” “parasternal intercostal plane block,” “PIP block,” and “fascia iliaca compartment block.” Priority was given to human pharmacokinetic studies, systematic reviews, experimental studies, and clinically relevant investigations.

In accordance with the recommendations of the RULE-AP framework,^8^ large language models were used during manuscript preparation to support medical English translation, grammar refinement, and figure generation. All scientific content, interpretations, critical appraisals, and final editorial decisions were performed and verified by the authors.

### Patient-related Factors

In FPBs, pharmacokinetics is influenced not only by the dose and physicochemical properties of the LA but also by patient-specific determinants that modulate absorption, distribution, metabolism, and elimination. The vascularity of the injected plane, total injectate volume, and protein binding characteristics of the drug interact with the patient’s physiological reserve and organ function to determine systemic exposure. Because FPBs often require relatively high volumes to achieve adequate distribution, systemic absorption becomes clinically relevant, thereby highlighting the importance of patient-related variability.^9^

Age is a major determinant of pharmacokinetic variability. Although reduced hepatic clearance and altered distribution in elderly patients are well recognized in general LA pharmacology, FPB-specific pharmacokinetic data in frail populations remain limited and are largely extrapolated from peripheral nerve and neuraxial block literature; among the few FPB-specific datasets, a pharmacokinetic study of fascia iliaca compartment block (FICB) (levobupivacaine 75 mg) in patients aged ≥80 years with hip fracture reported slow systemic absorption with all plasma concentrations remaining below the toxic threshold, suggesting that this technique is well tolerated in elderly patients despite the large injectate volume required.^10^ In addition, sarcopenia reduces the buffering capacity of skeletal muscle, which normally serves as a reservoir for circulating drugs. As muscle mass declines, redistribution toward toxicity-sensitive organs such as the brain and myocardium may occur more rapidly. Although some evidence supports a more conservative dosing approach in frail elderly patients, with practical dose reductions of approximately 10-20% suggested in the literature, FPB-specific dosing algorithms remain insufficiently validated. Accordingly, such reductions should be interpreted cautiously and individualized according to patient characteristics, procedural factors, and anticipated systemic exposure.^11^

In pediatric populations, pharmacokinetics varies significantly with developmental stage. Neonates and infants, particularly those younger than 6 months, have reduced plasma protein concentrations, including α1-acid glycoprotein (AAG) and albumin, which result in a higher unbound fraction of LAs. In addition, immature hepatic metabolism leads to reduced clearance and a prolonged elimination half-life. These factors collectively narrow the safety margin for systemic toxicity compared with adults.^12^ Clinical studies confirm that systemic absorption after FPBs is relevant in children: levobupivacaine administered via transversus abdominis plane (TAP) block reaches C_max_ values within approximately 20 minutes, with considerable interindividual variability, whereas ropivacaine concentrations following erector spinae plane block (ESPB) have, in some cases, exceeded neurotoxic thresholds defined in adults.^13, 14^ As children mature, pharmacokinetic parameters become more predictable; however, systemic exposure remains clinically significant.

The influence of biological sex on FPB pharmacokinetics is less well defined. Although FPB-specific evidence is limited, a recent volunteer study demonstrated that women had higher systemic exposure to ropivacaine despite similar block characteristics.^15^ However, this finding should be interpreted with caution. In that study, male participants were substantially heavier than female participants (median weights 80.0 vs. 62.5 kg), and both groups received an identical fixed dose of ropivacaine. Therefore, the higher exposure in women may partly reflect dosing relative to body weight rather than biological sex per se. Notably, the authors also attributed the difference to higher CYP1A2-mediated clearance in men. In contrast, other studies evaluating interfascial techniques such as ESPB and pectointercostal FPB have not identified a consistent relationship between pharmacokinetic parameters and demographic variables.^16^ Overall, sex alone appears to have limited clinical relevance, whereas associated factors, such as pregnancy, body composition, and comorbidity burden, are more important determinants.

Body composition plays a critical role in pharmacokinetics, whereas body mass index (BMI) alone is insufficient to guide dosing. In obese patients, total body weight increases disproportionately relative to lean body mass, and pharmacokinetic parameters such as volume of distribution and clearance do not increase linearly. Increased cardiac output and tissue perfusion may accelerate systemic absorption, while elevated AAG levels associated with chronic inflammation may partially buffer the unbound drug fraction.^17^ However, dosing based solely on total body weight, particularly in high-volume FPBs, may result in increased C_max _values and systemic exposure.^18^ Therefore, lean body weight, ideal body weight, or adjusted body weight provide a more appropriate basis for dosing. Recommendations regarding dosing adjustments in obesity are largely extrapolated from broader regional anaesthesia and pharmacokinetic literature, as FPB-specific evidence remains limited. Conversely, in underweight or catabolic patients, reduced muscle and fat reserves, impaired protein binding, and decreased clearance increase susceptibility to systemic toxicity.^11^ Thus, pharmacokinetics in FPBs is better explained by body composition and physiological reserve than by BMI alone.

Comorbid conditions, particularly hepatic, renal, and cardiac diseases, significantly influence pharmacokinetics. Amide LAs depend primarily on hepatic metabolism; therefore, their clearance may be reduced in advanced liver disease or conditions associated with decreased hepatic blood flow. Although dose adjustment may not be mandatory for single injections, a more conservative approach is warranted in the presence of repeated dosing, continuous infusions, or combined organ dysfunction. FPB-specific data support this approach: in patients undergoing major hepatectomy, high-dose bilateral TAP block has been associated with ropivacaine concentrations approaching toxic thresholds that are proportional to the extent of liver resection.^19^ Renal disease presents a more complex scenario. In severe renal failure, hyperdynamic circulation may lead to earlier C_max_ values, while increased AAG may partially reduce the unbound fraction. However, uremia and metabolic acidosis impair protein binding and increase toxicity risk, narrowing the safety margin.^20^ In cardiac disease, conduction abnormalities increase vulnerability to cardiotoxic effects, and reduced cardiac output limits hepatic and renal perfusion, further impairing drug elimination.

Pregnancy is one of the most significant patient-related risk factors. Much of the current understanding regarding pregnancy-related alterations in LA pharmacokinetics is extrapolated from neuraxial and peripheral nerve block literature rather than from FPB-specific investigations. Reduced concentrations of albumin and AAG increase the unbound fraction of LAs, while increased cardiac output and tissue perfusion enhance systemic absorption. Studies have demonstrated that the unbound fraction of bupivacaine increases progressively during pregnancy, particularly at term, thereby increasing the proportion of the drug within the toxic concentration range.^21^ Consequently, conservative dosing and vigilant monitoring are recommended for pregnant patients, particularly when high-volume interfascial blocks are performed.

Malnutrition, although poorly studied in FPBs, has clear pharmacokinetic implications. Altered body composition, reduced protein synthesis, and impaired organ function affect drug distribution and clearance. Low muscle mass and hypoproteinemia reduce buffering capacity and increase the variability of the unbound fraction.^22^ Although AAG is the primary binding protein for amide LAs, malnutrition is often associated with multiple physiological impairments, including reduced hepatic and renal function; this supports the use of lower doses and closer monitoring in these patients.

Clinically, patient-related factors determine key pharmacokinetic outcomes: the magnitude of systemic absorption, C_max_, unbound fraction, and elimination rate. Accordingly, individualized dosing strategies are essential in high-risk populations, including frail elderly patients, pregnant individuals, underweight or catabolic patients, and those with significant hepatic, renal, or cardiac disease. In conditions such as pregnancy, hypoalbuminemia, or renal dysfunction, reduced protein binding may increase the free fraction of circulating LA despite apparently acceptable total plasma concentrations. Furthermore, even apparently safe total plasma concentrations may be associated with systemic toxicity when they are accompanied by an increased free fraction of the LA, by acidosis, or by reduced protein binding. In such cases, conservative dosing, careful concentration-volume balance, fractionated injection, and close monitoring should be standard practice to minimize the risk of LAST. It should be acknowledged, however, that many of these recommendations are extrapolated from broader regional anaesthesia and LA pharmacology, as FPB-specific evidence for individualized dosing in high-risk populations remains limited.

### Local Anaesthetic-related Factors

The pharmacokinetic behavior of LAs in FPBs is largely determined by the interplay between drug-specific physicochemical properties and high-volume interfascial injection techniques.^9^ Given the marked variability in systemic absorption across different anatomical planes, intrinsic characteristics such as lipid solubility, protein binding, and metabolic profile play a central role in influencing both analgesic efficacy and toxicity risk. Accordingly, a clear understanding of drug-specific pharmacokinetics is essential for the safe and effective use of FPBs, in which systemic exposure following a bolus injection is typically characterized by C_max_ and T_max_.^9^

However, it should be emphasized that most clinical pharmacokinetic studies in FPBs report total plasma concentrations, whereas the free (unbound) fraction, which represents the portion available to interact with target tissues, correlates more directly with the risk of systemic toxicity.^9^ Commonly cited toxic plasma thresholds should therefore be interpreted with caution: the clinical likelihood of LAST is shaped not only by total plasma concentration but also by the free drug fraction, arterial versus venous sampling site, acid-base status, patient comorbidity, and individual susceptibility.^11^ A total concentration that appears acceptable by conventional standards may still produce toxicity when protein binding is diminished, the free fraction is elevated, or systemic acidosis is present.^9^

### Lidocaine

Beyond its LA effect, lidocaine has analgesic, anti-hyperalgesic, and anti-inflammatory properties that make it a versatile agent.^23^ Approximately 90% of the drug is metabolized in the liver; the elimination half-life is approximately 96 minutes.^23, 24^ Because lidocaine causes vasodilation, it is often absorbed rapidly from tissues; therefore, a vasoconstrictor is commonly added in clinical practice.^23^ Kato et al.^25^ measured serum lidocaine after bilateral TAP blocks with 400 mg total dose and found a mean C_max_ of 3.6±0.7 µg mL^-1^ at 30 minutes with individual peaks ranging from 2.7 to 5.5 µg mL^-1^. Absorption from ESP blocks is notably faster, with T_max_ values of 5-6 minutes.^26^ With continuous bilateral ESP catheters in pediatric cardiac surgery patients, Caruso et al.^27^ found mean peak lidocaine concentrations of 1.82 µg mL^-1^, well within the safe range. SAP catheter concentrations were approximately 43.5% higher than ESP values, likely due to differences in the administered dose.^28^ Although early toxicity signs are typically described at concentrations above 5 µg mL^-1^, and frank convulsions at concentrations above 10 µg mL^-1^, these reference values were largely derived from controlled intravenous studies and should therefore be applied to FPB settings with appropriate caution.^24^

### Bupivacaine

Bupivacaine’s prolonged action reflects its high lipophilicity (partition coefficient of 27.5), its pKa of 8.1, and extensive protein binding of 95-96%.^24, 29^ Reported toxicity thresholds, derived largely from volunteer infusion studies, are approximately 2.1 µg mL^-1^ for total venous concentrations and 4.0 µg mL^-1^ for arterial concentrations; these values should not be regarded as absolute, given the substantial influence of free fraction, sampling site, and individual susceptibility.^9, 30^ Notably, clinical toxicity has been documented even at doses below 2 mg kg^-1^.^9^

### Levobupivacaine

Levobupivacaine is the S(–)-enantiomer of bupivacaine, developed to retain anesthetic potency while reducing cardiac and neurological toxicity.^9, 31^ In FPBs, it is typically used at a concentration of 0.125-0.25%.^9^ Bardsley et al.^30^ reported that the maximum tolerated venous concentration was 2.62 µg mL^-1^ for levobupivacaine, compared with 2.25 µg mL^-1^ for bupivacaine, a small but clinically relevant difference. In ESP blocks using 2 mg kg^-1^ levobupivacaine, Shigeta et al.^32^ measured a C_max _of 1.24 µg mL^-1^ and a T_max_ of 6 minutes; epinephrine reduced the C_max_ by approximately half. Onishi et al.^33^ reported a median C_max_ of 553 ng/mL following levobupivacaine TAP block, approximately one-fifth of the commonly cited toxic threshold. Within the limits of currently available evidence, doses up to 2.5 mg kg^-1^ appear to be safe for FPBs.^9^

### Liposomal Bupivacaine

Liposomal bupivacaine employs DepoFoam^®^ technology, in which the drug is enclosed within multivesicular liposomes with a honeycomb-like internal structure that enables sustained release.^34^ This produces a characteristic bimodal plasma profile: an initial peak within the first hour, arising from the approximately 3% of free bupivacaine outside the liposomes, and a second peak at 12-36 hours as the encapsulated drug is gradually released.^35^ In volunteer studies, a fourfold-higher dose of liposomal bupivacaine produced nearly the same C_max _as plain bupivacaine (0.87 vs. 0.83 µg mL^-1^), while the terminal half-life was almost tenfold longer than that of plain bupivacaine.^34^ Importantly, the formulation should not be mixed with other LAs or come into contact with antiseptics such as chlorhexidine, since they disrupt the lipid membrane, causing rapid, uncontrolled drug release.^34^ At least 20 minutes should elapse between the injection of a different LA and the administration of liposomal bupivacaine.^34^

### Prilocaine

Prilocaine exhibits the weakest vasodilatory effect among amide LAs, permitting effective use without a vasoconstrictor.^23^ The combination of rapid clearance, low peak concentrations, and limited vasodilation makes prilocaine an appealing candidate, at least in theory, for high-volume FPBs, although direct pharmacokinetic data in FPB settings remain limited.^36^

### Ropivacaine

Ropivacaine was the first long-acting LA marketed as a pure S(–)-enantiomer, and its lower lipophilicity provides a wider safety margin for both CNS and cardiovascular toxicity than bupivacaine.^31, 37^ It also shows relative motor sparing, with smaller Aδ and C fibers being blocked more readily than large motor fibers.^37^ Approximately 94% of circulating ropivacaine is protein-bound to AAG, and it is hepatically metabolized by CYP1A2 and CYP3A4, with approximately 86% of metabolites excreted renally.^37^

In FPB studies, ropivacaine is usually prepared at concentrations of 0.25-0.5% in volumes of 20-40 mL.^9^ When doses approach 3 mg kg^-1^ in TAP blocks, mean total venous concentrations reach 1.8-2.0 µg mL^-1^, and some patients exceed the commonly cited toxicity threshold of 2.2 µg mL^-1^; however, free concentrations were not consistently measured in these studies.^9^ Toju et al.^38^ reported a mean arterial C_max_ of 1.87±0.78 µg mL^-1^ after subcostal TAP at 3 mg kg^-1^. PECS blocks appear relatively concerning: Versyck et al.^39^ observed that half of their 20 patients had free ropivacaine concentrations in the potentially toxic range at 2.5 mg kg^-1^, a finding that is particularly informative because the unbound fraction was directly quantified. In a population pharmacokinetic analysis of continuous ESP blocks, Schwenk et al.^40^ found that 5 of 15 patients reached plasma concentrations approaching reported toxic thresholds despite standard dosing; their model predicted that halving the initial bolus from 150 mg to 75 mg would substantially reduce this risk. Clinicians should also be aware that CYP1A2 inhibitors, such as fluvoxamine, can elevate ropivacaine levels and may necessitate a dose adjustment.^37^ Because FPBs are frequently performed under general anaesthesia, which can mask the early CNS signs of toxicity, close hemodynamic monitoring for at least 30-60 minutes after injection and immediate availability of lipid emulsion for rescue remain important safety measures.^38, 41^

Overall, LA pharmacokinetics in FPBs reflect a complex interplay among drug properties, injection-site vascularity, total dose, and patient factors.^5, 9, 42^ Although the literature continues to grow, one consistent observation is that plasma concentrations may approach or exceed commonly cited toxic thresholds even when conventional weight-based dose limits are respected, particularly with bilateral or high-volume techniques.^9, 42^ In clinical practice, this argues for a more conservative approach to high-volume bilateral truncal FPBs, particularly in frail elderly patients, parturients, and those with hepatic dysfunction or hypoalbuminemia. Using lower concentrations at adequate volumes may reduce systemic exposure while preserving interfascial spread.^9, 11^ Current dosing recommendations and agent-specific safety considerations relevant to FPBs are summarized in Table 1.^9, 11, 23, 24, 25, 26, 30, 32, 34, 35, 36, 37, 39, 40, 43, 44^ These values should be interpreted with caution. Reported toxic plasma thresholds predominantly reflect total venous concentrations derived from heterogeneous studies, including controlled volunteer infusions, and may not directly translate to clinical FPB practice, where free drug fraction, sampling methodology, acid-base status, and patient comorbidities all influence individual risk. Although current evidence supports cautious dose reduction in vulnerable populations (e.g., the elderly, parturients, and patients with hepatic dysfunction or hypoalbuminemia), precise FPB-specific dosing algorithms remain insufficiently validated. Population pharmacokinetic modeling, such as the ropivacaine model developed by Schwenk et al.,^40^ offers a promising path toward more individualized, site-specific dosing. Moving forward, prospective studies with serial plasma sampling across different block types and patient populations, ideally including measurement of free drug fractions, will be needed to replace empirical dose ceilings with genuinely evidence-based guidelines.^26, 42^

### Adjuvant-related Factors

In FPBs, adjuvants are commonly used to enhance analgesic efficacy and prolong block duration. From a mechanistic perspective, adjuvants can be broadly classified into two categories: (i) agents that modify the pharmacokinetics of LAs and (ii) agents that primarily enhance analgesia through pharmacodynamic mechanisms without substantially altering LA pharmacokinetics. Pharmacodynamic effects primarily involve modulation of nociceptive transmission, inflammatory signaling, and neuronal excitability, whereas pharmacokinetic effects involve alterations in LA absorption, systemic uptake, tissue distribution, or plasma concentration profiles. Importantly, several adjuvants likely exert combined effects through both pathways, and the relative contribution of each mechanism remains incompletely defined (Table 2).^1, 45, 46, 47^ This distinction is particularly relevant because FPBs differ fundamentally from conventional peripheral nerve blocks. In FPBs, drugs are injected into relatively large interfascial compartments rather than directly adjacent to a discrete nerve. Consequently, injected solutions may undergo substantial redistribution within fascial planes, diffusion into adjacent muscular compartments, and variable vascular absorption depending on the anatomical characteristics of the block. These factors significantly influence systemic drug exposure and complicate the interpretation of whether observed prolongation of analgesia is attributable to local peripheral mechanisms, systemic effects, or a combination of both.

### Epinephrine: Modulation of Absorption Kinetics

Epinephrine is the most established pharmacokinetic adjuvant in regional anaesthesia. Its primary mechanism is local vasoconstriction, which reduces vascular uptake and modifies the systemic absorption profile of LAs. However, in FPBs, the magnitude of this effect is highly dependent on the anatomical characteristics of the injected fascial plane, including vascularity, compartment size, and diffusion dynamics.^1, 48^

Across truncal FPBs, the most consistent pharmacokinetic effect of epinephrine is prolongation of T_max_, indicating a slower absorption rate. In contrast, its effect on C_max_ is less predictable and appears to be block-specific. For example, in TAP blocks, epinephrine reliably delays T_max_ but does not consistently reduce C_max_, likely because the relatively large, compliant fascial compartment facilitates widespread distribution.^48, 49^ Conversely, in more confined spaces such as the rectus sheath, epinephrine may reduce both T_max_ and C_max_, reflecting a more pronounced limitation of systemic uptake.^50^

These findings highlight that the pharmacokinetic behavior of epinephrine in FPBs is not uniform but depends on the interaction between vasoconstriction and fascial plane characteristics. Clinically, epinephrine should therefore be considered primarily as a safety-enhancing agent that delays systemic absorption rather than a reliable means of reducing overall systemic exposure or consistently prolonging analgesia.^48, 49, 50^ Its use may be particularly valuable in high-volume truncal blocks, where large LA doses increase the risk of systemic toxicity.

### Dexmedetomidine: Dual Local and Systemic Effects

Dexmedetomidine (DEX) is one of the most extensively studied adjuvants in FPBs and exhibits both pharmacodynamic and pharmacokinetic interactions. Evidence indicates that DEX undergoes clinically relevant systemic absorption when administered in fascial planes, with measurable plasma concentrations following TAP block.^46^ In addition, numerous clinical studies report sedation, bradycardia, hypotension, and opioid-sparing effects consistent with systemic α2-adrenergic receptor activation.^45, 46, 47,51^

In ESPB and TAP block studies, DEX has been associated with a prolonged duration of analgesia and a delayed requirement for rescue analgesics. Although these effects are frequently attributed to local peripheral mechanisms, the observation of dose-dependent systemic effects, including sedation and hemodynamic changes, suggests that systemic absorption may also contribute to its clinical profile.^45, 46, 47, 51^ Accordingly, the analgesic effects of DEX in FPBs likely reflect a combination of peripheral modulation of nociceptive transmission and systemic pharmacodynamic effects following vascular uptake, although the relative contribution of each mechanism remains incompletely understood.

The potential of DEX to reduce LA absorption via local vasoconstriction has been proposed, but pharmacokinetic evidence supporting this effect in FPBs remains inconsistent.^1, 45, 52^ Consequently, the prolongation of analgesia observed with DEX is more plausibly explained by pharmacodynamic mechanisms than by substantial modification of LA pharmacokinetics.^1, 45^

Current clinical evidence suggests that DEX may be an effective adjuvant in selected FPBs. In the TAP block, randomized controlled trials demonstrate prolonged analgesia and reduced opioid consumption, while in the ESPB, both RCTs and meta-analyses show delayed time to first rescue analgesia and decreased opioid requirements.^45, 46, 47, 51^

Dose-dependent adverse effects are clinically relevant. Lower doses (e.g., 0.5 µg/kg) provide analgesic benefits with a more favorable hemodynamic profile, whereas higher doses (e.g., 1 µg/kg) prolong analgesia further but increase the incidence of bradycardia and hypotension.^47, 53^ Given its systemic absorption, caution is required in hemodynamically unstable or vulnerable patients.

The prolongation of analgesia observed with DEX likely reflects a combination of peripheral and systemic pharmacodynamic effects, whereas its influence on LA absorption remains inconsistent across studies.^45, 46, 47,51^ Although local vasoconstrictive effects have been proposed, current pharmacokinetic evidence does not consistently demonstrate substantial reductions in systemic LA exposure.

### Dexamethasone: Pharmacodynamic Enhancement

Dexamethasone is among the most commonly used adjuvants in regional anaesthesia and has been associated with prolonged analgesia in multiple studies. Its mechanism in FPBs is primarily pharmacodynamic, involving anti-inflammatory effects, reduced nociceptor sensitization, and attenuation of rebound pain.^45, 54^

Unlike epinephrine, dexamethasone does not significantly reduce LA absorption through vasoconstriction. Instead, its benefits appear to result from modulation of the inflammatory milieu and peripheral nociceptive pathways. Clinical studies and meta-analyses have demonstrated prolonged analgesia and reduced opioid consumption when dexamethasone is added to TAP or ESP blocks.^55, 56^

Combination strategies, particularly the combination of dexamethasone and DEX, have recently attracted increasing interest in FPBs because several studies have reported prolonged analgesia and improved postoperative recovery profiles compared with single-adjuvant techniques.^55, 56, 57^ Some investigations have also suggested potential modulation of perioperative stress and inflammatory responses with these combinations.^56, 57^ However, the currently available evidence remains limited by substantial heterogeneity in study design, block type, adjuvant dosing, and outcome assessment. In addition, the relative contributions of peripheral pharmacodynamic mechanisms and systemic absorption remain insufficiently defined. Although additive or synergistic interactions are biologically plausible, the pharmacokinetic consequences of combined adjuvant administration within interfascial compartments have not yet been adequately characterized. Therefore, combination adjuvant strategies should be interpreted cautiously until larger FPB-specific pharmacokinetic and pharmacodynamic studies become available.

### Magnesium Sulfate: NMDA-mediated Modulation

Magnesium sulfate (MgSO_4_) acts primarily through NMDA receptor antagonism, reducing central sensitization and nociceptive transmission. In FPBs, its role appears to be predominantly pharmacodynamic, as it does not consistently alter LA pharmacokinetics or systemic absorption.^58, 59^

Clinical evidence, particularly from TAP block studies, suggests that MgSO_4_ can prolong analgesia and reduce postoperative opioid consumption.^58^ However, its efficacy is less consistent than that of α_2_ agonists or corticosteroids, and results vary across block types and study designs.^60, 61^ In ESPB, its effects may be present but are often less pronounced than those observed with DEX.^60, 61^

Magnesium generally has a favorable safety profile, but systemic absorption may occur after high-volume FPBs. Excessive exposure can theoretically lead to hypotension, sedation, or neuromuscular weakness, particularly in patients with renal dysfunction or impaired magnesium homeostasis.^60, 61, 62^ Thus, while MgSO_4_ may be a useful alternative adjuvant, its role remains less well-defined.

### Opioid-derived Adjuvants: Peripheral vs. Systemic Effects

Opioid-derived adjuvants, including buprenorphine, tramadol, and nalbuphine, have been investigated in regional anaesthesia for their potential to prolong analgesia via peripheral opioid receptor activation. However, in FPBs, it is often difficult to distinguish local receptor-mediated effects from those resulting from systemic absorption.^63^

Given the relatively large volumes used in FPBs and the variable vascularity of fascial planes, systemic uptake of these agents may be substantial, leading to effects similar to those of intravenous administration.^1, 5, 16^ Clinical studies comparing tramadol and DEX in the TAP block demonstrate differences not only in analgesic efficacy but also in adverse-effect profiles, including sedation and nausea, which are more likely attributable to systemic exposure.^64^ Similarly, combination regimens such as nalbuphine with DEX in ESPB highlight the increasing interest in multimodal adjuvant strategies.^65^

From a pharmacokinetic perspective, the systemic effects of opioid-derived adjuvants may limit their advantage as purely peripheral agents. Adverse effects such as sedation and postoperative nausea and vomiting are likely driven by systemic concentrations, reinforcing the need for cautious dosing and patient selection.^63, 64, 65^

### Synthesis: Efficacy versus Pharmacokinetic Safety

The literature on FPBs reflects considerable heterogeneity in both efficacy and mechanisms. While some reviews emphasize their effectiveness within multimodal analgesia, others highlight variability in clinical outcomes.^66, 67, 68, 69^ This divergence is largely attributable to the inherent variability of FPBs, which depend on fascial plane anatomy, injectate spread, and systemic absorption dynamics.^1, 16, 52^

Accordingly, adjuvant selection should not be based solely on the expectation of prolonged analgesia. Instead, it should incorporate an assessment of pharmacokinetic safety, particularly in high-volume truncal blocks where systemic exposure may be substantial. The key question is whether a given adjuvant will enhance analgesia without narrowing the safety margin by increasing systemic exposure or potentiating adverse effects.^6, 16^

### Clinical Approach to Adjuvant Selection

A rational approach to adjuvant use in FPBs should consider three principal factors: the characteristics of the fascial plane, the desired duration of analgesia, and patient-specific risk factors. First, the anatomical plane determines the absorption kinetics. Blocks associated with rapid systemic uptake require greater emphasis on safety, including conservative LA dosing and consideration of epinephrine to modulate absorption.^16, 39^ Although current evidence supports cautious dose reduction in vulnerable populations, precise FPB-specific dosing algorithms remain insufficiently validated. Pharmacokinetic studies in different FPBs demonstrate substantial variability in systemic exposure depending on block type and patient characteristics.^10, 18^

In practical terms, a risk-stratified approach may be preferable to fixed dosing strategies. Lower initial LA doses, incremental injection techniques, and consideration of vasoconstrictive adjuvants such as epinephrine may be reasonable in patients at increased risk of systemic toxicity, including elderly individuals, patients with reduced hepatic or renal reserve, patients at extremes of body weight, or patients with conditions associated with altered protein binding.^10, 17, 18, 19, 20, 21^ However, these recommendations are primarily extrapolated from broader regional anaesthesia principles, as validated FPB-specific dosing frameworks remain limited. Second, the desired duration of analgesia should guide adjuvant selection. For shorter procedures, optimization of LA dose and volume may be sufficient. For prolonged analgesia, a single well-established adjuvant—most commonly dexamethasone or DEX—may be preferred over multiple agents.^45, 47, 51, 53, 57^ Third, patient-related risk factors must be integrated into decision-making. Patients prone to hemodynamic instability may not tolerate DEX well, while those with diabetes may experience systemic corticosteroid effects. Magnesium should be used cautiously in patients with renal impairment.^58, 60, 62^

### Fascial Plane-related Factors

Peripheral nerve blocks and FPBs differ fundamentally in their pharmacokinetic behavior. In conventional nerve blocks, LA is deposited adjacent to a defined neural structure, resulting in relatively predictable spread and absorption. In contrast, FPBs involve the injection of larger volumes into interfascial compartments, where drug distribution and systemic uptake depend on fascial architecture, tissue vascularity, and dynamic fluid behavior within the plane.^1, 52^

Fascia consists of a collagen-rich matrix embedded in a hydrated environment containing glycosaminoglycans, adipocytes, and fibroblasts. While the dense collagen network restricts macroscopic fluid flow, it permits diffusion of small molecules such as LAs through microscopic channels. Following injection, the LA distributes along the fascial plane, diffuses into adjacent tissues, and is absorbed into the systemic circulation. The relative contribution of these pathways varies according to the anatomical location and local perfusion. Because FPBs require larger injectate volumes, systemic absorption may be greater than that observed with traditional nerve blocks.^1, 52^

Beyond anatomical and physiological determinants, the pharmacokinetic profile of an FPB is also strongly influenced by operator-dependent factors. Because ultrasound-guided FPBs rely on accurate identification of the correct interfascial plane and on real-time visualization of needle-tip position during injection, even subtle differences in technique (needle-tip depth), injection pressure, the pattern of hydrodissection, and unintended intramuscular or subcutaneous deposition can alter the surface area of fascia exposed to LA, the rate of capillary absorption, and ultimately the systemic concentration profile.^3, 66^*In vivo* observations and microdialysis measurements have shown that interstitial and plasma concentrations may vary substantially even when identical doses are injected by experienced practitioners, underscoring the inherent variability of these techniques.^1^

The TAP block is one of the most extensively studied FPBs and illustrates key pharmacokinetic principles. In this technique, LA is injected between the internal oblique and transversus abdominis muscles, targeting thoracolumbar nerve branches. This plane is relatively well vascularized by intercostal and deep circumflex iliac vessels, which contribute to systemic uptake.^70^ C_max_ values typically occur within 20-60 minutes, with reported ropivacaine concentrations ranging from approximately 0.4 to 1.6 µg mL^-1^, depending on dose and technique.^50, 71^ Although these levels generally remain below toxicity thresholds, the available evidence suggests that bilateral blocks and continuous infusions may lead to higher systemic exposure. In obstetric patients, bilateral TAP blocks have been associated with plasma concentrations approaching toxic levels, and significant interindividual variability has been observed during continuous infusions.^71, 72^ These findings support individualized dosing strategies in high-volume FPBs.

The rectus sheath block (RSB), another abdominal wall technique, exhibits a similar pharmacokinetic profile, albeit with delayed absorption. When performed with comparable volumes and concentrations, RSB yields C_max_ values similar to those of the TAP block but with a longer T_max_, likely reflecting differences in compartment characteristics and vascularity.^73^

Quadratus lumborum block (QLB) targets the fascial compartments surrounding the quadratus lumborum muscle and the thoracolumbar fascia. Although several approaches exist, pharmacokinetic data suggest that C_max_ values generally remain below toxic thresholds.^74^ The addition of epinephrine has been shown to reduce systemic absorption and prolong analgesia, further illustrating the interaction between pharmacokinetics and fascial plane characteristics.^75^

Thoracic FPBs are often performed in highly vascular regions, resulting in relatively rapid systemic absorption. The ESP block, located adjacent to the dorsal branches of intercostal and lumbar arteries, demonstrates rapid uptake, with T_max_ values frequently occurring within 5-10 minutes.^32^ Despite this rapid absorption, reported C_max_ values of levobupivacaine typically remain below toxic thresholds, usually around 1-1.5 µg mL^-1^.^10, 50^ This pattern suggests that rapid uptake does not necessarily translate into toxic exposure, but underscores the importance of considering both the rate and the extent of absorption.

A recent systematic review of pharmacokinetic studies of single-injection ESP block identified nine eligible studies with amide LAs and confirmed that a short T_max _is a near-universal feature of the technique: in most studies, the peak concentration coincided with the earliest sampling time point, and pharmacokinetic modelling estimated T_max_ as early as 1 minute after injection.^76^ The same review reported that eight patients in two studies exceeded total LA toxicity thresholds, although in most cases this was driven by methodological choices (venous rather than arterial sampling, or the use of conservative threshold definitions). More importantly, only two of the included studies measured free (unbound) plasma concentrations: in four pediatric patients receiving 1.5 mg kg^-1^ ropivacaine, the peak unbound arterial concentration exceeded the central nervous system toxicity threshold of 0.6 µg mL^-1^ as defined by Knudsen et al.^77^ even though total concentrations remained below accepted limits; however, this finding is based on only four paediatric patients in a pilot pharmacokinetic study judged at unclear risk of bias, and should therefore be interpreted as hypothesis-generating rather than definitive. In adults, the median free bupivacaine concentrations were approximately 20-fold lower than reported toxicity thresholds.^76^

Similarly, interpectoral and pectoserratus plane blocks, performed in the pectoral region, are associated with rapid systemic uptake due to the dense vascular network supplied by thoracoacromial, lateral thoracic, and intercostal arteries. C_max_ values are generally reached within 10-30 minutes, although total systemic exposure typically remains within safe limits.^39, 78^

The serratus anterior plane (SAP) block further illustrates the influence of vascularity and dose on pharmacokinetics. Performed in a highly vascular region, the SAP block demonstrates relatively rapid absorption, with C_max_ values typically occurring within 10-20 minutes and ranging from 1.5 to 2.0 mg/L, depending on dose and concentration.^79, 80^ In pediatric cardiac surgery, plasma concentrations around 2.1 mg/L have been reported without signs of systemic toxicity.^81^

The deep parasternal intercostal plane (PIP) block generally results in lower systemic exposure despite bilateral administration. Mean peak bupivacaine concentrations have been reported around 0.6 µg mL^-1^ following doses up to 2.5 mg kg^-1^. However, two patients had concentrations >2.0 µg mL^-1^ within 15 minutes of the block. The injection site is located close to the internal mammary artery.^82^

Taken together, these data indicate that FPBs fall along a spectrum of systemic absorption, which has direct implications for monitoring and dosing. At the higher-absorption end lie the thoracic and chest-wall techniques: the ESP block shows the most rapid uptake, with T_max_ frequently occurring within the first few minutes and modelled peaks estimated as early as one minute, while the SAP and interpectoral/pectoserratus blocks similarly reach C_max_ within 10-30 minutes, reflecting the dense vascularity of these regions. At the lower-absorption end are the abdominal wall blocks (TAP, rectus sheath, and QLB), in which C_max_ typically occurs later (approximately 20-60 minutes for the TAP block and later still for the RSB), and peak concentrations at equivalent doses tend to be lower.^50, 71, 73, 74^ However, a rapid T_max_ does not necessarily imply a higher C_max_; for example, after ESP blocks, rapid uptake is usually accompanied by peak concentrations below accepted thresholds. In addition, the extent of absorption still matters at the tails of the distribution, as illustrated by the PIP block, in which mean exposure is low yet individual patients exceeded 2.0 µg mL^-1^ within 15 minutes,^82^ likely reflecting proximity to the internal mammary artery.

Clinically, this spectrum translates into a graded approach to monitoring and dose selection. Thoracic blocks with higher absorption warrant vigilance in the early post-injection period and the use of the lowest effective concentration, together with consideration of epinephrine in high-volume single-injection techniques; the slower-absorbing abdominal wall blocks provide a somewhat wider temporal margin but still require caution when performed bilaterally or at high volume. Because the highest plasma concentrations after slower-absorbing or continuous techniques may occur well beyond the intraoperative period, the duration of monitoring should be extended accordingly.

The FICB is performed to reach the nerves of the lumbar plexus distally with a single injection.^83^ FICB demonstrates that systemic exposure may vary substantially depending on technique, patient population, and dosing strategy. Studies of early FICB using landmark techniques in pediatric patients, employing 0.7 mL/kg of 0.375% or 0.5% ropivacaine reported peak ropivacaine concentrations as high as 4.3-5.6 µg mL^-1^, raising concerns about systemic toxicity.^84^ In a more recent study, none of the patients had total or free ropivacaine levels above the toxic concentration after suprainguinal FICB with 40-mL of 0.5% ropivacaine.^85^ These findings highlight the importance of technique optimization and dose adjustment, particularly in vulnerable populations.

An additional determinant of pharmacokinetic variability in FPBs is the heterogeneity of drug distribution within fascial compartments. Microdialysis studies have shown that interstitial LA concentrations may vary significantly within the same plane, even across small distances.^86^ This uneven distribution implies heterogeneous neural exposure to effective drug concentrations, contributing to variability in analgesic efficacy and systemic absorption.

The pharmacokinetics of continuous catheter infusions warrant separate consideration. A review of TAP and paravertebral catheters found that manufacturer-recommended dosing limits were exceeded in 28% of ropivacaine infusions and 51% of bupivacaine infusions, with LAST occurring in 0.48% and 0.37% of patients, respectively.^87^ These data suggest that, for continuous catheter techniques, the cumulative 24-hour dose may be a more clinically relevant determinant of toxicity risk than the size of any single bolus.^26^ The main risk factors identified were a high 24-hour cumulative dose, bilateral catheters, cardiac surgery, use of cytochrome P-450 inhibitors, and hypoalbuminemia.^87, 88^ Continuous infusions also tend to produce higher plasma concentrations than programmed intermittent boluses at equivalent total doses,^42^ and there is very little pharmacokinetic data available for infusions running longer than 20 hours.^26^ Unlike single-injection FPBs, continuous interfascial infusions introduce an additional risk of delayed accumulation and a progressive rise in plasma concentration over several days, particularly when bilateral catheters are used or clearance is impaired.^26^ In bilateral SAP catheters with programmed intermittent bolus delivery, Chen and Tsui^28^ found that lidocaine plasma concentrations peaked around days 2-3 at a mean concentration of approximately 2.6 µg mL^-1^, with considerable variability between patients (SD 32-44%), highlighting the importance of ongoing monitoring during prolonged infusions.

A pragmatic, risk-stratified approach is useful when translating these pharmacokinetic data into clinical practice. FPBs can be stratified by their expected systemic absorption profile: thoracic blocks typically produce a rapid Tmax and should be regarded as higher absorption techniques, whereas TAP, RSB, and QLB show slower absorption and lower peak concentrations at equivalent doses.^42^ Patient-level risk should be assessed. On this basis, the following principles, rather than fixed algorithms, may be suggested: (i) calculate the total LA dose on a weight-adjusted basis, using ideal body weight in cases of obesity; (ii) apply a downward adjustment of approximately 25-30% in identified high-risk patients and in any bilateral or continuous technique; (iii) prefer the lowest effective concentration over the lowest effective volume when reducing dose, in order to preserve spread; and (iv) consider the addition of epinephrine in high-volume single-injection thoracic FPBs. These suggestions are presented as expert-opinion recommendations rather than validated dosing algorithms, which remain an unmet need.

Explicit monitoring after high-volume FPBs is warranted because the highest plasma concentrations may not coincide with the operating-room phase. After single-injection bilateral or high-volume FPBs, continuation of standard ASA monitoring is recommended throughout the block and for at least 30-60 minutes thereafter in a monitored environment, with immediate availability of resuscitation equipment and 20% lipid emulsion in accordance with current LAST management guidelines.^11, 89^ For continuous interfascial infusions, monitoring should extend beyond the immediate postoperative period, and ward staff should be educated to recognize early symptoms of LAST.

Despite growing clinical use, pharmacokinetic data for many FPBs remain limited. In particular, few studies report unbound (free) plasma concentrations, which are more directly associated with systemic toxicity than total drug levels. Furthermore, variability in study design, sampling protocols, and patient characteristics complicates comparisons across techniques. Future research should focus on standardized pharmacokinetic methodologies, the routine reporting of unbound drug concentrations, and improved characterization of dose-concentration relationships across different fascial planes, with the goal of developing prospectively validated, FPB-specific dosing and monitoring frameworks.

### Heterogeneity in the Literature

Interpretation of the current pharmacokinetic literature on FPBs is substantially limited by heterogeneity in study methodology, block techniques, dosing strategies, sampling protocols, and analytical reporting standards.^5, 9, 37, 42^ As a result, direct comparisons between studies are often difficult and may lead to overinterpretation of apparent differences in systemic absorption or toxicity risk.

One of the major sources of heterogeneity relates to variability in LA dosing and injection volume. Studies investigating TAP, ESPB, SAP, and QLB frequently use different concentrations, total doses, and bilateral versus unilateral injection strategies, all of which may substantially influence reported plasma concentrations.^5, 9, 13, 14, 16, 25, 38, 40, 48, 49, 75^ For example, some TAP block studies reported peak plasma ropivacaine concentrations approaching potentially toxic thresholds, whereas others reported substantially lower systemic exposure despite using similar techniques.^5, 9, 13, 25, 38^ However, these findings are not directly comparable because the studies differed considerably in total dose administered, injection volume, patient body habitus, and timing of blood sampling.

Similarly, variability in pharmacokinetic sampling methodology represents another important limitation. Reported C_max_ and T_max_ values may be highly dependent on plasma-sampling frequency and duration.^9, 42^ Studies employing sparse sampling protocols may fail to identify true peak concentrations, particularly in FPBs, where systemic absorption may continue over prolonged periods because of the large interfascial surface area.^5, 9, 26^ Consequently, differences in reported C_max _values between studies may reflect methodological variation rather than true pharmacokinetic differences.

Additional heterogeneity arises from inconsistencies in reporting total versus free plasma LA concentrations. Some studies report only total plasma concentrations, whereas others additionally measure unbound (free) fractions, which may correlate more closely with systemic toxicity risk.^6, 9, 11, 37, 44^ Variability in assay methodology and reporting standards therefore complicates interpretation of toxic thresholds and limits the ability to establish universally applicable pharmacokinetic safety margins across FPBs.^6, 9, 12, 42^

Comparisons between different FPBs are similarly challenging. Apparent differences in systemic absorption between TAP, ESPB, PECS, SAP, and QL blocks may reflect not only anatomical differences in fascial plane vascularity but also substantial methodological heterogeneity among studies.^1, 2, 3, 52, 66, 67, 90^ Factors such as needle approach, exact injectate location, spread pattern, patient positioning, ultrasound guidance technique, and operator experience may influence systemic uptake and clinical outcomes.^1, 2, 3, 52, 66, 67, 90^ Furthermore, the anatomical continuity of some fascial planes may facilitate unpredictable injectate spread, thereby increasing variability in both pharmacokinetic and analgesic findings.^1, 52, 90^

Continuous catheter studies introduce an additional layer of complexity. Continuous infusion protocols vary considerably in background infusion rate, intermittent bolus strategy, cumulative LA dose, and infusion duration.^27, 28^ These differences substantially influence systemic exposure and make cross-study comparisons particularly difficult. In addition, accumulation kinetics during prolonged interfascial infusion remain poorly characterized in many FPBs.^26, 27, 28^

Substantial heterogeneity also exists in patient populations included in current studies. Age, body composition, hepatic function, renal function, pregnancy status, and perioperative hemodynamic conditions may all influence LA disposition and systemic absorption.^12, 15, 17, 18, 19, 20, 21, 22, 42^ For instance, elderly patients and individuals with reduced plasma protein binding capacity may exhibit altered pharmacokinetics and increased susceptibility to systemic toxicity despite similar administered doses.^10, 17, 22^

The same limitations apply to studies investigating adjuvants. Considerable variability exists in adjuvant dosing, combinations, timing of administration, and outcome definitions. In DEX studies, some investigators evaluate fixed doses, whereas others use weight-based dosing regimens, complicating the interpretation of dose-response relationships and systemic effects.^45, 46, 47, 51, 53, 57^ Likewise, studies examining dexamethasone, MgSO_4_, ketamine, or combination adjuvant strategies differ substantially in methodology, which makes pooled interpretation difficult.^45, 54, 55, 56, 57, 58, 59, 60, 61, 62^

Importantly, many studies focus primarily on analgesic outcomes without an integrated pharmacokinetic analysis. Consequently, prolonged analgesia is often interpreted as evidence of local interfascial mechanisms, despite limited assessment of systemic drug exposure. This distinction is particularly relevant for adjuvants such as DEX, clonidine, opioid-derived agents, MgSO_4_, and ketamine, in which clinically relevant systemic absorption may contribute significantly to observed analgesic effects.^45, 46, 47, 51, 53, 54, 55, 56, 57, 58, 59, 60, 61, 62, 63, 64, 65^

Taken together, the current FPB literature should be interpreted cautiously. Although existing studies provide valuable insights into interfascial analgesia and adjuvant use, substantial methodological heterogeneity limits definitive conclusions regarding comparative pharmacokinetics, toxicity thresholds, and mechanistic interpretations. Future investigations would benefit from standardized reporting of block technique, LA dosing, plasma sampling protocols, free versus total drug concentrations, and clinically relevant pharmacokinetic-pharmacodynamic correlations.

### Local Anaesthetic Systemic Toxicity

LAST is a serious complication of regional anaesthesia and is recognized in the European Resuscitation Council 2025 guidelines as a “special circumstance” due to its distinct pathophysiology and management.^91^ The American Society of Regional Anesthesia and Pain Medicine defines LAST as systemic toxicity resulting from elevated plasma concentrations of LAs, caused by inadvertent intravascular injection or significant systemic absorption, leading to CNS and/or cardiovascular dysfunction.^6^

The clinical presentation of LAST is variable and may involve neurological, cardiovascular, or combined manifestations. Traditional descriptions suggest an initial phase of CNS excitation—such as perioral numbness, tinnitus, agitation, or seizures—followed by CNS depression and subsequent cardiovascular compromise. However, systematic analysis of published cases demonstrates that this sequence is not universal.^92^ Contemporary review literature also notes that, with the increasing use of truncal and interfascial techniques, atypical and delayed toxicity patterns are increasingly being recognised, and early neurological warning signs may be masked in sedated or anaesthetised patients.^93^

In FPBs, the risk of LAST is closely linked to pharmacokinetic characteristics. While ultrasound guidance reduces the likelihood of intravascular injection, it does not eliminate the risk of toxicity.^6, 93^ Pharmacokinetic studies demonstrate that high-volume interfascial injections can result in measurable systemic absorption, with plasma concentrations in some cases approaching toxic thresholds.^5^ Therefore, LAST in FPBs may arise not only from direct intravascular injection but also from significant systemic uptake.

Macfarlane et al.^93^ noted that a growing proportion of LAST reports now arise from fascial-plane and field blocks, with delayed-onset presentations becoming increasingly common, partly because ultrasound guidance reduces but does not eliminate inadvertent intravascular injection, and partly because early neurological warning signs may be masked in sedated or anaesthetised patients. Pharmacokinetic studies demonstrate that plasma concentrations, in some patients, approach or exceed established toxicity thresholds, even in the absence of overt clinical symptoms.^5, 6^

Reported cases of LAST associated with TAP blocks illustrate the risks inherent in high-volume bilateral techniques. Griffiths et al.^71^ documented symptomatic neurotoxicity in postpartum patients receiving bilateral ropivacaine TAP blocks (2.5 mg kg^-1^) after caesarean section, with peak plasma ropivacaine concentrations of 2.2-3.8 µg mL^-1^ within 60 minutes; neurological symptoms resolved spontaneously without intravenous lipid emulsion (ILE). Boleyn et al.^89^ reported LAST-induced cardiac arrest following bilateral TAP block with liposomal bupivacaine in a 68-year-old man undergoing open sigmoidectomy, successfully treated with advanced cardiac life support and 20% ILE. Regarding asymptomatic threshold exceedances, Hessian et al.^72^ found that four of 20 patients exceeded the accepted threshold for total ropivacaine concentration (3.4 mg/L) during 72-hour bilateral TAP infusions without neurological symptoms, highlighting the dissociation between total plasma concentration and clinical toxicity. Torup et al.^94^ similarly demonstrated that bilateral TAP blocks with 20 mL of 0.5% ropivacaine per side (total 200 mg) produced potentially toxic peak total concentrations (above 2.2 µg mL^-1^) in six of 18 patients (33%); one patient experienced a 33% drop in mean arterial pressure, although none showed overt LAST. In a patient with cardiac, respiratory, and renal dysfunction, Ishida et al.^95^ documented peak total ropivacaine concentrations of 2.5 µg mL^-1^ at 15 minutes following bilateral TAP blocks with only 1.8 mg kg^-1^, a potentially neurotoxic level despite a conventionally low dose; the patient became drowsy but recovered without intervention.

ESPB-related LAST cases span both adult and neonatal populations and encompass single-injection and continuous catheter techniques. Yawata et al.^96^ described neurological LAST (dizziness, speech disturbance, and loss of consciousness) within minutes following 30 mL of 0.5% levobupivacaine (150 mg, 2.6 mg kg^-1^) for lumbar spinal surgery in a 58 kg patient; symptoms resolved with supportive care without ILE. Crowe and Mislovič^97^ reported the first neonatal LAST case following ESPB: a 6-day-old, 4.0 kg neonate developed acute cardiovascular compromise (falling oxygen saturation, hypotension) within minutes of receiving 1 mg kg^-1^ levobupivacaine for laparotomy, successfully treated with epinephrine and ILE 1.5 mL/kg. In continuous-catheter settings, Smith et al.^98^ described delayed-onset LAST from an ESPB lidocaine catheter in a post-cardiac surgery patient, in whom toxicity manifested several days postoperatively due to impaired hepatic lidocaine clearance secondary to liver shock from worsening pulmonary hypertension; this illustrates that organ dysfunction can convert a standard infusion rate into a toxic cumulative dose.

Pharmacokinetic studies further demonstrate that subclinical threshold exceedances are not rare in FPB practice. In a systematic review of TAP block and RSB pharmacokinetics, Rahiri et al.^5^ found that mean C_max_ values were generally below accepted toxicity thresholds but that individual patients exceeded these limits in several studies, particularly with high-volume bilateral techniques. In a systematic review of single-injection ESPB pharmacokinetics, De Cassai et al.^76^ reported that eight patients across two studies exceeded total LA toxicity thresholds. Importantly, in the two studies that measured free (unbound) plasma concentrations, paediatric patients receiving 1.5 mg kg^-1^ ropivacaine had peak unbound arterial concentrations exceeding the CNS toxicity threshold of 0.6 mg/L, even when total concentrations remained within conventional limits, whereas adult free bupivacaine concentrations remained well below toxic levels.^14^ Bailey et al.^9^ synthesised pharmacokinetic data across multiple FPB types and concluded that while mean plasma concentrations of ropivacaine, levobupivacaine, and bupivacaine typically remain below established thresholds, a consistent minority of patients exceed accepted toxicity levels without overt LAST symptoms; risk is substantially amplified by fixed-volume dosing, higher LA concentrations, bilateral or continuous infusions, and patient comorbidities such as pregnancy, extremes of age, and hepatic or cardiac disease. Collectively, these data confirm that total plasma concentration alone is an insufficient safety surrogate: patients with reduced protein binding or impaired clearance may have a clinically relevant free drug fraction substantially higher than their total concentration would suggest.

Management requires immediate, structured intervention (Table 3). Current guidelines recommend cessation of LA administration, airway management with optimization of oxygenation and ventilation, and prompt seizure control.^6, 91^ Correction of hypoxia and acidosis is essential, as both exacerbate toxicity.^6^ In cases of cardiovascular instability or cardiac arrest, advanced life support should be initiated, recognizing LAST as a toxin-mediated condition.^91^ ILE therapy is a cornerstone of treatment for severe toxicity.^6, 91^

ILE is believed to reverse severe LAST through complementary pharmacokinetic and pharmacodynamic mechanisms rather than through a single “lipid sink” effect.^99, 100, 101, 102^ The most widely accepted framework is the dynamic lipid shuttle, whereby an expanded intravascular lipid phase partitions highly lipophilic LAs away from toxicity-sensitive organs such as the heart and brain, lowers their freely available concentration at these target sites, and promotes their redistribution through the circulation to the liver, skeletal muscle, and adipose tissue for subsequent storage, metabolism, and detoxification.^99, 100, 102^ In parallel, ILE appears to exert direct cardiocirculatory effects by enhancing both inotropy and lusitropy, thereby improving myocardial contractile and relaxation performance, supplying fatty-acid substrate for ATP generation, restoring intracellular calcium homeostasis, attenuating mitochondrial dysfunction and permeability transition pore opening, and partially counteracting toxic LA induced vasoplegia and conduction abnormalities through modulation of nitric oxide-dependent vasorelaxant signaling and, at least in part, sodium-channel dysfunction.^99, 100, 101, 103^ At the vascular and electrophysiologic levels, ILE may also partially reverse LA-induced vasodilatory responses, plausibly through modulation of nitric oxide-dependent vasorelaxant signaling, and attenuate ion-channel dysfunction, particularly sodium-channel blockade, thereby contributing to restoration of vascular tone, improvement in conduction stability, and earlier hemodynamic stabilization.^99, 100, 101, 103^

## Conclusion

FPBs represent a paradigm shift in regional anaesthesia, characterized by variable and often significant systemic absorption due to high-volume interfascial injection. Pharmacokinetic variability appears to be shaped by patient-, drug-, fascial plane-, technical-, and adjuvant-related factors, each influencing C_max_, T_max_, and ultimately toxicity risk (Table 4). In particular, advanced age, impaired protein binding, highly vascular interfascial planes, bilateral or continuous techniques, and higher total doses may increase systemic exposure and susceptibility to LAST. Interpretation of the current pharmacokinetic literature is further complicated by substantial heterogeneity in study design, including differences in LA dose, concentration, sampling intervals, assay methodology, patient populations, and reporting of total versus free plasma concentrations. Therefore, a patient-centered approach that integrates dose optimization, appropriate adjuvant selection, and vigilant monitoring is essential. Detailed clinical guidance on the pharmacokinetics and safety of FPBs is presented in Supplementary Material 1. Future research should focus on standardized pharmacokinetic models and harmonized reporting strategies to enable more precise, evidence-based dosing recommendations in FPBs.

## Supplementary Materials

Supplementary Material https://d2v96fxpocvxx.cloudfront.net/cf9d60d6-523c-458a-a2e6-78728d3ffbb0/content-images/ba894618-953a-4603-859f-dbaaecbd6d07.pdf

## Figures and Tables

**Table 1. Suggested Dose Considerations and Agent-Specific Pharmacokinetic Features for Local Anesthetics used in FPBs:** 

**Agent**	**MRD plain (mg kg^-1^)**	**MRD+Epi (mg kg^-1^)**	**FPB-specific considerations**
Lidocaine	5 (350 mg)	7 (500 mg)	Rapid absorption from ESP (T_max_ 5-6 min); bilateral TAP C_max_ can reach 5.5 µg mL^-1^; early toxicity signs reported above 5 µg/mL; vasoconstrictor often added in clinical practice
Bupivacaine	2 (175 mg)	3 (225 mg)	LAST reported at doses <2 mg kg^-1^; narrowest seizure-to-cardiac-arrest margin; reported venous toxicity threshold ~2.1-2.25 µg mL^-1^; consider avoiding continuous infusions where feasible (51% of infusions exceeded MRD)
Levo-bupivacaine	2 (200 mg)	3 (225 mg)	≤2.5 mg kg^-1^ appears safe for FPBs; epinephrine halves C_max_ in ESP block; reported maximum tolerated venous concentration 2.62 µg mL^-1^; less cardiotoxic than racemic bupivacaine
Ropivacaine	3 (200 mg)	3 (250 mg)	Suggested limits of ≤2 mg kg^-1^ without epinephrine and ≤2.5 mg kg^-1^ with epinephrine for FPBs; reported venous toxicity threshold ~2.2 µg mL^-1^; 50% of patients had free ropivacaine concentrations in the potentially toxic range in PECS blocks at 2.5 mg kg^-1^; caution with CYP1A2 inhibitors; motor-sparing at low concentrations
Prilocaine	6 (400 mg)	8 (600 mg)	Fastest clearance (2.84 L min^-1^); no vasoconstrictor needed; theoretical advantage for high-volume FPBs; dose-dependent methemoglobinemia risk (contraindicated in sickle cell disease); limited FPB-specific PK data
Liposomal bupivacaine	266 mg max*	-	Bimodal PK profile (peaks at ~1 h and 12-36 h); do not mix with other LAs or antiseptics; wait ≥20 min after conventional LA injection; ~4× dose produces similar C_max_ as plain bupivacaine

These values should be interpreted cautiously, as most recommendations are derived from narrative reviews and heterogeneous pharmacokinetic data rather than formal dose-finding studies. MRD, maximum recommended dose; Epi, epinephrine; FPB, fascial plane block; ESP, erector spinae plane; TAP, transversus abdominis plane; PECS, pectoral nerves; PK, pharmacokinetic; LA, local anesthetic; LAST, local anesthetic systemic toxicity;

C_max_, peak plasma concentration. *Not weight-based; absolute dose limit. Traditional MRD values adapted from El-Boghdadly et al.^11^ and Rosenberg et al.^44^; FPB-specific recommendations from Bailey et al.^9^

**Table 2. Pharmacokinetic Effects of Adjuvants in Fascial Plane Blocks:** 

**Adjuvant**	**Principal mechanism in FPBs**	**Effect on LA absorption kinetics**	**Expected effect on T_max_**	**Expected effect on C_max_**	**Evidence consistency/certainty**	**Practical clinical interpretation**
Epinephrine	Local vasoconstriction	Decreases absorption rate	↑ Often prolonged	↓ Variable; may be unchanged in some planes and reduced in others	Moderate consistency; effects on C_max_ and analgesic duration remain block-dependent	Primarily considered when modulation of systemic absorption and widening of the PK safety margin are desired rather than direct analgesic enhancement
Dexmedeto-midine	Local antinociceptive effect+systemic α_2_-mediated action after absorption	Variable effect on LA absorption; not consistently demonstrated across all planes	Variable	Variable	Moderate but heterogeneous evidence; systemic absorption likely contributes to clinical effects	May be considered when prolonged analgesia is desired, although caution is required in hemodynamically vulnerable patients
Dexametha-sone	Anti-inflammatory and antinociceptive action	Minimal direct effect on LA absorption	Usually no major direct PK effect	Usually no major direct PK effect	Moderate consistency for analgesic prolongation, although FPB-specific PK mechanisms remain insufficiently characterized	Reasonable option for prolonging analgesia without prominent α_2_-mediated hemodynamic effects
Magnesium sulfate	NMDA receptor antagonism	Minimal direct effect on LA absorption	Usually unchanged	Usually unchanged	Limited-to-moderate evidence with substantial interstudy heterogeneity	Primarily a pharmacodynamic adjuvant rather than a PK modifier
Tramadol	Peripheral and systemic analgesic contribution	No consistent major effect on LA PK	Usually unchanged	Usually unchanged	Limited and heterogeneous evidence	May be considered when α_2_ agonists are undesirable, but systemic adverse effects should be considered
Nalbuphine/opioid-derived adjuvants	Peripheral opioid receptor effect + systemic contribution	No consistent major effect on LA PK	Usually unchanged	Usually unchanged	Limited FPB-specific evidence	Potential utility remains uncertain because peripheral and systemic contributions are difficult to distinguish

C_max_, peak plasma concentration; FPB, fascial plane block; LA, local anesthetic; NMDA, N-methyl-D-aspartate; PK, pharmacokinetic; T_max_, time-to-peak concentration

**Table 3. Structured Management Approach to Local Anaesthetic Systemic Toxicity:** 

**Clinical stage**	**Recommended management**	**Dose/monitoring**
Initial response	Immediately stop local anaesthetic administration. Call for help. Obtain LAST rescue kit. Consider cardiopulmonary bypass team. Consider early lipid emulsion therapy.	—
Lipid emulsion therapy (20%)-adults >70 kg	Administer intravenous lipid emulsion.	Bolus 100 mL over 2-3 min, followed by infusion 250 mL over 15-20 min. If instability persists, repeat bolus and double infusion rate.
Lipid emulsion therapy (20%)-<70 kg	Administer intravenous lipid emulsion.	Bolus 1.5 mL kg^-1^ over 2-3 min, followed by infusion 0.25 mL kg^-1^ min^-1^. Consider infusion pump if <40 kg. Repeat bolus if instability persists. Maximum cumulative dose 12 mL kg^-1^.
Seizure management	Ensure airway protection. Administer benzodiazepine (e.g., lorazepam 0.1 mg kg^-1^ IV, midazolam 0.05-0.1 mg kg^-1^ IV). If only propofol available, use small incremental doses (e.g., 20 mg).	Avoid large doses in cardiovascular instability.
Cardiovascular instability (arrhythmia /hypotension)	LAST resuscitation differs from standard ALS. Use reduced-dose epinephrine. Avoid additional local anaesthetics, vasopressin, beta-blockers, and calcium channel blockers.	Start epinephrine ≤1 µg kg^-1^.
Post-stabilisation monitoring	Continue lipid infusion for at least 15 min after haemodynamic stability.	Observe ≥2 h after seizure; 4-6 h after cardiovascular instability; longer after cardiac arrest.

ALS, advanced life support; LAST, local anaesthetic systemic toxicity; IV, intravenous

**Table 4. Determinants of Pharmacokinetics in Fascial Plane Blocks:** 

**Factor category**	**Specific factor**	**Mechanism affecting pharmacokinetics**	**Effect on C_max_**	**Effect on T_max_**	**Clinical implications in FPBs**
Patient-related factors	Age (elderly)	↓ Hepatic/renal perfusion, ↓ clearance, ↓ muscle reservoir	↑	↔/↑	Accumulation risk, dose reduction (≈10-20%)
-	Pediatrics (especially <6 months)	↓ Protein binding, immature metabolism	↑ (free fraction ↑)	↑	Narrow safety margin, cautious dosing
-	Pregnancy	↓ Albumin and AAG, ↑ cardiac output	↑ (free fraction ↑)	↓	Increased systemic absorption, toxicity risk
-	Body composition (obesity)	↑ Cardiac output, altered distribution	↑ (if TBW-based dosing)	↓	Dose based on lean/adjusted weight
-	Low body mass/malnutrition	↓ Protein binding, ↓ tissue buffering	↑	↔	Higher free drug fraction, toxicity risk
-	Hepatic disease	↓ Metabolism of amide LAs	↑ (with repeated dosing)	↔	Accumulation, cautious use in infusions
-	Renal disease	Altered protein binding, acidosis	↑ (free fraction ↑)	↓	Increased toxicity susceptibility
-	Cardiac disease	↓ Perfusion → ↓ clearance	↑	↔	Increased cardiotoxic risk
LA-related factors	Lipid solubility	↑ Tissue penetration, slower redistribution	↑	↑	Prolonged effect, higher toxicity risk (e.g., bupivacaine)
-	Protein binding (AAG)	↓ Free fraction when high	↓ (free fraction)	↔	Higher binding=longer duration, safer profile
-	Metabolic pathway	Hepatic vs. extrahepatic metabolism	↑ (if impaired metabolism)	↔	Drug-specific accumulation risk
Adjuvant-related factors	Epinephrine	Vasoconstriction → ↓ vascular uptake	↓ (variable)	↑	Reduces the risk of systemic toxicity
-	Dexmedetomidine	Systemic absorption + α_2_ effect	Variable	Variable	Prolonged analgesia, HD effects
-	Dexamethasone	Anti-inflammatory (PD effect)	↔	↔	Prolonged analgesia
-	Magnesium	NMDA antagonism	↔	↔	Prolonged analgesia, PD contribution
-	Opioid-derived adjuvants	Systemic absorption	↔	↔	Effects often systemic rather than local
Fascial plane-related factors	Vascularity of plane	Determines systemic uptake rate	↑ in highly vascular planes	↓	Faster absorption (e.g., ESP, PECS)
-	Plane compliance/ volume	Larger space → wider distribution	↑ (dose-dependent)	↑	High-volume techniques increase systemic exposure and toxicity risk
-	Bilateral blocks	Increased total dose	↑	↔	Higher systemic exposure
-	Continuous infusion	Drug accumulation over time	↑ progressively	↔	Increased LAST risk
-	Injection technique (bolus vs. infusion)	Alters absorption kinetics	↑ (continuous infusion)	↔	Intermittent bolus may be safer
Technical factors	Total dose (mg)	Primary determinant of systemic exposure	↑	↔	Most critical predictor of toxicity
-	Volume vs. concentration	Volume affects spread, dose affects C_max_	↑ (with total dose)	↑ (volume-dependent)	Balance required for efficacy vs. safety
-	Injection rate	Faster injection → higher peak levels	↑	↓	Fractionated injection recommended

AAG, alpha-1-acid glycoprotein; ESP, erector spinae plane; FPB, fascial plane block; HD, hemodynamic; LA, local anesthetic; LAST, local anesthetic systemic toxicity; PD, pharmacodynamic; PECS, pectoral; TBW, total body weight; C_max_, peak plasma concentration; T_max_, time to peak plasma concentration; NMDA, N-methyl-D-aspartate
